# Soldiers’ resilience? A systematic review of micro-, meso-, and macro-level resilience factors in the military

**DOI:** 10.3389/fpsyg.2026.1857328

**Published:** 2026-07-22

**Authors:** Gloria Ch. Straub, Wolfgang H. Prinz, Brigitte Lueger-Schuster

**Affiliations:** 1 Federal Ministry of Defence, Vienna, Austria; 2 Department of Clinical and Health Psychology, Faculty of Psychology, University of Vienna, Vienna, Austria; 3 Vienna Doctoral School in Cognition, Behavior and Neuroscience (VDS CoBeNe), Vienna, Austria

**Keywords:** high-risk occupations, military, military culture, multilevel perspective, occupational health, resilience, soldiers, systematic review

## Abstract

The military profession is highly challenging because soldiers are exposed to potentially traumatic stressors which cannot be avoided. Given that these stressors are associated with psychological health risks, the need for resilience has become crucial within the military. Although research has recognized the importance of a multilevel consideration of resilience in military populations, little research has been done on this so far. Hence, we aimed to systematically review individual (micro-level) and organizational (meso- and macro-level) resilience factors among active-duty soldiers. During our search, we identified nine studies for inclusion, of which two used qualitative and seven applied quantitative methods. We identified 82 resilience factors on all levels. Most studies focused on micro-level factors, such as adaptive coping or individual characteristics, whereas meso- (e.g., leadership behavior) and macro-level factors (e.g., structural conditions) remain underrepresented. Moreover, no included study investigated multilevel factors with nested data. We discuss these findings from a systemic perspective, shifting the responsibility for building a resilient environment from the individual to the organization. Thus, we highlight that resilience should be promoted through significant organizational practices that ensure access to resources, enhance a sense of belonging, and reduce stigma. Moreover, we recommend that military organizations first address structural conditions and cultural barriers to resilience. Our findings contribute to a more comprehensive understanding of how resilience can be developed across micro, meso, and macro levels within military organizations and offer new perspectives for sustainable resilience building in the military.

**Systematic review registration:**

https://www.crd.york.ac.uk/prospero/, identifier [CRD42021254629].

## 1 Introduction

In addition to traditional low-risk stressors (e.g., work overload; Brooks and Greenberg, 2018; Campbell and Nobel, 2009), soldiers are often exposed to high-risk stressors (e.g., exposure to high-risk situations, witnessing injuries/death) that can include potentially traumatic events (Adler and Castro, 2013; Flood and Keegan, 2022; Van Hooff et al., 2014). These additional stressors are inherent to their profession and thus cannot be avoided or minimized. However, work-related stress has become a major challenge for soldiers (Britt et al., 2016a), as it can be associated with mental health problems (Bonde et al., 2016; Killgore et al., 2006; Pietrzak et al., 2012).

Research on work-related stress outcomes has been characterized by significant activity and substantial contributions, whereas the exploration of stress-protective factors has been comparatively limited (Sefidan et al., 2021). Additionally, the role of psychological resilience (henceforth referred to as resilience) has been recognized as crucial in high-risk occupations, such as the military (Sun et al., 2024).

Our study aimed to systematically review resilience among soldiers by focusing on individual (micro level) and organizational (meso- and macro-level) resilience factors among active-duty military personnel. We first provide an overview of resilience with a focus on the military and highlight the necessity for a multilevel framework. Further, we outline inconsistencies in resilience research and explore the need for a narrow definition of resilience.

### 1.1 Conceptualizing resilience in the military

Despite a large body of research, the literature conceptualizes resilience heterogeneously and lacks a coherent definition (Luthar et al., 2000; Masten, 2001; Meredith et al., 2011; Pangallo et al., 2015). According to Chmitorz et al. (2018), resilience can be defined in three ways: as a trait, an outcome, or a process. Britt et al. (2016b) comprehensively integrate these three definitions within an organizational framework. They distinguish between the amount of resilience that resides within an individual (capacity for resilience) and the adaption of relevant outcomes (demonstration of resilience). The former is influenced by micro-, meso-, and macro-level resilience factors (e.g., personality traits, cohesion) or processes (e.g., coping, help-seeking). The latter refers to outcomes of resilience (e.g., performance, health).

A multilevel approach (Davydov et al., 2010; Windle, 2011), in which resilience factors range from the macro (e.g., society, organization) to the meso (e.g., peer group) to the individual micro layer (e.g., psychological), is particularly crucial for high-risk organizations, such as the military because they are organized hierarchically, and individuals serve together in teams within units.

The need for a multilevel approach to investigating resilience in the military is highlighted in a recent conceptual review (Polusny and Erbes, 2023). Little attention has been paid to multilevel resilience factors within this population thus far. To our knowledge, only one review has addressed resilience factors in this context (Meredith et al., 2011). Therefore, it seems timely to provide a systematic overview of the literature on resilience factors at different levels within the military.

### 1.2 The need for a narrow definition of resilience

The literature uses the term resilience in a broad way, which is problematic as is creates difficulties in comparing the definitions, operationalizations, measures, and results (Kalisch et al., 2015). Within a broad framework, resilience is often equated with related concepts (e.g., hardiness, post-traumatic growth) and not specified as an independent construct. Although an overlap between resilience and related constructs exists, the latter are distinct from resilience and defined differently (Wald et al., 2006; Windle, 2011). In addition, when framing the term broadly, being resilient or not can be defined binarily as the absence or presence of psychopathology (e.g., Sarapas et al., 2011). Consequently, their predictors will be identical but opposite in direction (Bonanno and Diminich, 2013).

To compare resilience in terms of its definitions, operationalizations, measures, and results, we conceptualized it as an independent construct. We defined it as “the capacity of a dynamic system to adapt successfully through multisystem processes to challenges that threaten the function, survival, or development of the system” (Masten et al., 2021, p. 524) and distinguished it from the demonstration of resilience. We further characterized it as a capacity that encompasses individual (micro), interpersonal (meso), and environmental (macro) promotive factors. Although we used a systemic perspective, we focused exclusively on resilience factors within an organizational system, in our case, the military organization. Therefore, in contrast to Bergström and Dekker (2014), the macro level does not refer to societal factors but to the military community, and the meso level describes factors within units and teams. In line with Bergström and Dekker (2014), the micro level pertains to the individual. We henceforth refer to those factors as micro-, meso-, and macro-level resilience factors.

### 1.3 The present study

Our study aimed to investigate the capacity for resilience in active-duty soldiers. First, we identified different resilience factors by synthesizing the scientific literature. Second, we classified these factors by disentangling the micro, meso, and macro levels of military personnel and the military environment.

To gain a better understanding of the specific needs of soldiers and promote a deeper insight into how to enable a resilient military organization, we formulated the following research questions: RQ1. Which factors on an individual (micro) level promote the psychological resilience of soldiers according to the scientific literature? RQ2. Which factors on a military unit (meso) level promote the psychological resilience of soldiers according to the scientific literature? RQ3. Which factors on a military community (macro) level promote the psychological resilience of soldiers according to the scientific literature?

## 2 Methods

This pre-registered study used the method of a systematic review (PROSPERO-ID: CRD42021254629; 15/06/2021) and was conducted according to the PRISMA guidelines (Page et al., 2021). Supplementary File H provides the PRISMA Checklist. All members of the review team (GCS, WHP, BLS) are clinical psychologists and experienced in the current research field. GSC and WHP are military psychologists.

### 2.1 Search strategy

Given the substantial conceptual heterogeneity within resilience research, we restricted the search to studies that explicitly investigated resilience and resilience factors rather than broader constructs that are associated with resilience. To enhance conceptual comparability across studies and to ensure consistency with our definition of resilience as a distinct capacity rather than an outcome, we excluded studies that defined resilience as the absence of psychopathology, focused on related constructs (e.g., hardiness) or evaluated training programs with heterogeneous intervention components.

The first author (GCS) systemically searched the PubMed, PsycINFO, SCOPUS, and Web of Science databases up to May 2025 using the following terms and their synonyms: (a) resilience, AND (b) factor, AND (c) military. The searches were tailored for each database; truncations and asterisks were used where appropriate. Supplementary File A provides the full search strategy.

The database search was limited to peer-reviewed studies written in either English or German that were published since 2000. To identify additional studies, GCS hand-searched the reference lists of all the included studies and undertook forward citation tracking using Google Scholar.

Prior to the main searches, GCS conducted preliminary searches of all four databases and screened the results to ensure that the search terms were precise. The results were reviewed and discussed by the research team to assess the relevance and specificity of the search terms, resulting in a refined search strategy for the main review.

### 2.2 Study selection

**FIGURE 1:**
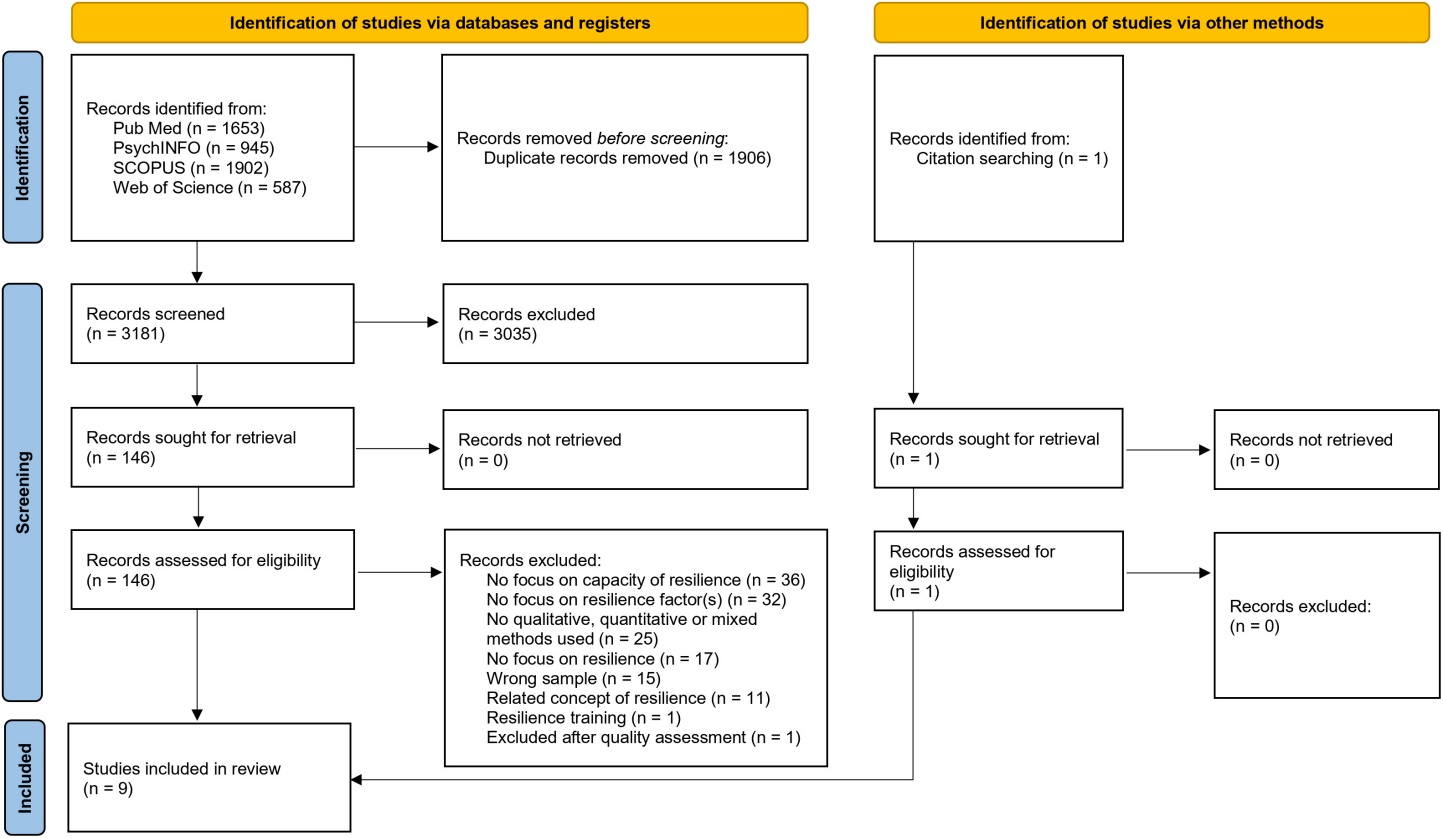
PRISMA flow chart.

The PRISMA flow (Figure 1) illustrates the study selection process. Guided by the PICOS framework (see Supplementary File B), the initial selection was based on the following inclusion criteria: (a) peer-reviewed journal article, (b) qualitative, quantitative, or mixed-methods study, (c) military sample, (d) participants aged 18 years or older, (e) focused on the capacity for resilience, and (f) addressed resilience factor(s) on at least one level.

For comparison reasons, we limited the scope to active-duty soldiers; this was because they serve as full-time professionals with unique organizational and environmental conditions. We did not consider studies on reservists, reserve duty, or National Guard soldiers. Moreover, we excluded veteran samples, which are defined heterogeneously in the literature and often used as convenience samples only (Lira and Chandrasekar, 2020).

Record screening of title, abstract, and keywords and full-text assessment were conducted by GCS. Records for which eligibility could not be determined with confidence were retained for full-text assessment. When information, which was required for an eligibility decision, was missing from the full-text, the corresponding authors were contacted for clarification. During full-text screening, cases of uncertainty regarding the application of the inclusion and exclusion criteria were discussed with the co-authors until consensus was reached.

Duplicates were detected automatically using the reference management tool Zotero. They were then manually confirmed by GCS as records that were the same and excluded.

### 2.3 Data extraction and analysis

Relevant information regarding the study, participants, research design, resilience factors, and capacity for resilience was transferred into *a priori* developed data extraction spreadsheets. Missing data were requested from the authors of the studies. The qualitative and quantitative studies were then analyzed separately. A meta-analysis was not feasible due to heterogeneity across studies regarding resilience factors, study designs, and measure instruments.

For the qualitative data, we used thematic analysis, a method that identifies, analyses, and reports patterns within qualitative data (Braun and Clarke, 2006). GCS conducted the initial analysis by searching the extracted data for resilience factors, grouping them at the micro, meso, or macro level, and creating initial themes. Factors were assigned according to their primary source and mechanism of influence. When appropriate, some resilience factors were categorized simultaneously to more than one level. The categorization was then evaluated by the whole research team. Disagreement was resolved through group discussion. After editing the pattern of meaning, groups, and themes, the revised version was discussed and refined again by the research team. Finally, the initial themes were transferred to key themes. Coding rules, theme definitions, and examples are provided in Supplementary File C.

For the quantitative data, all the extracted resilience factors were grouped at either the micro, meso, or macro level and assigned to the key thematic analysis themes by GCS. Again, the whole team reviewed the process. In case of divergence, the groupings were discussed and revised until team agreement was achieved.

## 3 Results

The database search identified a total of 5,087 records (3,181 without duplicates). After screening their titles, keywords, and abstracts, 146 full texts were assessed for eligibility, resulting in nine records remaining for final analysis. Reference list search led to one additional paper.

Studies that met the eligibility criteria were assessed for their quality and risk of bias independently by GCS and WHP using the Cohort Study Checklist (Critical Appraisal Skills Programme, 2018a), the Qualitative Studies Checklist (Critical Appraisal Skills Programme, 2018b), and the Critical Appraisal Checklist for Analytical Cross Sectional Studies (Joanna Briggs Institute, 2020). Any ambiguity was discussed with the senior researcher (BLS). Six studies fulfilled nearly all appraisal criteria and were considered as high quality, while three studies contained minor methodological uncertainties, primarily related to the reporting of confounding factors and insufficient reporting of follow-up duration and completeness. One study was excluded. Detailed item-level assessments of the included studies are provided in the Supplementary File D. The records that were excluded after full-text screening and quality assessment are provided in Supplementary File E.

### 3.1 Study characteristics

All the included studies were written in English and published between 2016 and 2024. Seven used quantitative and two qualitative methods. Abraham et al. (2018) collected data from a longitudinal mixed-methods study. However, their analysis only dealt with qualitative cross-sectional data, which is why we labeled the study as qualitative in our review. Most of the studies had cross-sectional designs (88.9%) and more than half were from North America (55.6%). More details of the study characteristics can be found in Supplementary File F. Given the predominance of cross-sectional study designs, no causal conclusions can be drawn from the available evidence. Therefore, the findings are reported as associations or perceived resilience factors rather than factors that promote resilience.

Except for one paper with no particular definition of resilience (Doody et al., 2022), all the studies theoretically defined resilience and focused on its capacity. Although Doody et al. (2022) did not provide a definition, they qualitatively explored a concept of resilience that aligned well with the focus of our review, which is why we included the paper.

### 3.2 Sample characteristics

Our review included active-duty personnel from six different armed forces. Most of the studies were from the United States Armed Forces (44.4%) and investigated Army (44.4%) and/or Navy (33.3%) populations; 33.3% involved enlisted ranks and officers, whereas 22.2% included enlisted ranks only. Our review was limited to active-duty soldier populations. However, Charbonneau (2019) focused solely on military cadets. To clarify the sample and decide whether the paper was eligible for inclusion, we corresponded with the author (02/2024). She advised that all participants were military students at the Royal Military College of Canada and active military members, which is why we included the paper in our review. Three studies did not report any rank or pay grade information. Table 1 describes the characteristics of the samples in the included studies. Additional characteristics are available in the data extraction form (see Supplementary File G).

**TABLE 1:** Characteristics of the samples.

ID	References	Military	Sample	Subsample	*N*	Age M SD [Range]	% males	% rank	Years in service M SD [Range]
^1^	Adler et al. (2023)	US Armed Forces	Soldiers	A	6,284	n.r. n.r. [n.r.]	80.4	57.4 Jr. enl. 28.5 NCO 13.5 O	n.r. n.r. [n.r.]
^2^	Cai et al. (2017)	People’s Liberation Army	Soldiers	n.r.	1,477	21.3 3.3 [17–38]	100	93 Enl. 7 O	3.0 3.1 [1–20]
^3^	Charbonneau (2019)	Canadian Armed Forces	Military undergraduates	N, A	328	20.4 3.5 [17–36]	79	100 Cadets	n.r. n.r. [n.r.]
^4^	Reyes et al. (2020)	Philippine Army	Military personnel	n.r.	360	33.2 6.9 [20–53]	90.8	n.r.	10.6 6.4 [1–32]
^5^	Rice and Liu (2016)	US Armed Forces	Military personnel	A, AF, N, M, CG	63	53.1 11.2 [24–74] ^a^	50.8	n.r.	15.4 8.6 [1–34]
^6^	Valladares-Garrido et al. (2022)	Peruvian Armed Forces	Military personnel	n.r.	586	n.r. n.r. [19–32]	94	n.r.	n.r. n.r. [n.r.]
^7^	Yurgil et al. (2024)	US Armed Forces	Soldiers	M, N	1,835	22.4 3.3 [n.r.]	100	100 Jr. enl.	n.r. n.r. [n.r.]
^8^	Abraham et al. (2018)	US Armed Forces	Combat medics	A	17	28.0 6.4 [n.r.]^b^	74^b^	79 Jr. enl. 20 NCO^b^	6 5.2 [n.r.]^b^
^9^	Doody et al. (2022)	Irish Defence Forces	Soldiers, officers	A, N, AF	12	32 5.7 [24–40]	83	41.6 Jr. enl. 8.3 NCO 50 O	11.5 5.7 [3–20]

US, United States; n.r., not reported; A, Army; N, Navy; AF, Air Forces; M, Marines; CG, coast guards; Jr. enl., junior enlisted; NCO, non-commissioned officer; O, officer; Enl., enlisted.

^a^Calculated for the total sample containing active-duty personnel and veterans.

^b^Calculated for the baseline sample (*N* = 840).

The sample size in the qualitative studies ranged from 12 to 17 participants. Their reported mean age ranged from 28 to 32 years, and they were primarily male. The sample size in the quantitative studies ranged from 63 to 6,284 participants. Their reported mean age ranged from 20.4 to 53.1 years. Five studies investigated male and female participants; the other two papers only included males. Except for one study (Rice and Liu, 2016), the majority of participants were men.

### 3.3 Qualitative studies

The two qualitative studies used in-depth and semi-structured interviews for data collection and applied grounded theory analysis and thematic analysis to explore the capacity for resilience. In the present review, we analyzed the extracted data; our findings are presented in Table 2.

**TABLE 2:** Results of the thematic analysis–assignments of the resilience factors from qualitative studies to key themes.

Coding	Resilience factors
**Micro level**	
Theme: interpersonal resources	
Experience	Interpersonal experiences on social bonding behavior^8^
Personal traits and skills	Networking/maintaining a supportive social network^9^
Theme: intrapersonal resources	
Adaptive coping skills	Active coping skills^8,9^, compartmentalization^9^, emotion regulation^8^, emotionally detaching^9^, maintaining alertness^8^, maintaining focus^8^, physical activity^9^, physical coping^9^, staying physically well^9^
Personal traits/skills	Altruism^8^, being present^8^, empathy by supporting others^8^, following their instincts^8^, optimism^9^, reflection skills^9^, social bonding behavior^8^, team working skills^8^, thinking independently^8^
Experience	Experience in their role^9^, experience of similar critical incidents^9^, general life experience^9^
Theme: knowledge	
Awareness of the military	Awareness of the military culture^8^
Experience training	Acquiring skills and traits necessary for first combat experience^8^
Knowledge sharing	Exchanging/sharing knowledge and skills^9^
Theme: military identity	
Attitude	Advocating for their soldiers^8^, expressing pride in the work^9^, feeling of making a difference and doing good^9^, just doing the job^8^, seeing the bigger picture^9^
Readiness	Following their instincts^8^, military enlisting as destiny^8^
**Meso level**	
Theme: social environment	
Leadership behavior	Destigmatizing leading^9^, leading and managing subordinates^9^, leading by caring for others^8^, leading by example^8^, leading by informing transparently^9^, leading dutifully/in a conscientious way^8^, leading in a respectful way^8^, leading through commitment^8^, leading through integrity^8^
Camaraderie and companionship	Acting as a military family^9^, cohesion^9^, companionship between leaders and subordinates^8^, companionship btwn. soldiers^8^, social bonding (with the crew)^ 9^, unit climate^9^
Social connectedness	Cohesion^9^
Social support	Exchanging/sharing knowledge and skills^9^
Theme: training	
Informal training	Exchanging/sharing knowledge and skills^9^
**Macro level**	
Theme: knowledge	
Certainty of information	To be informed^9^
Theme: organizational framework	
Structural conditions	Availability and access to support service (during mission)^9^, equality btwn. soldiers^8^, leave periods^9^, mandatory rest periods^9^, military standard procedures^9^, rest and recuperation^9^
Theme: training	
Psychological skill training	Compartmentalization training^9^, predeployment training with psychoeducational approach to emotional reactions^9^, resilience training containing exposure to critical incidents and desensitization^9^, resilience training containing mental resilience tools^9^, resilience training with psychoeducational approach to resilience^9^, self-confidence training^9^, self-efficacy training^9^
Military training	Early career training containing military^9^, initial medic training with real-life or closest to real-life experience^8^, predeployment training containing exposure to highly realistic critical incident scenarios^9^, predeployment training with technical, job-related content^9^, prophylactic training in stressful situations (vaccination) containing exposure to critical incidents and desensitisation^9^, scenario-based training^9^

Superscript numbers indicate which study reported the factor. The number refers to the study ID, which is provided in Table 1.

We identified 33 individual (micro)-level resilience factors that are linked with the resilience of soldiers (RQ1); most frequently, we assigned them to the theme intrapersonal resources, where the majority were coded as adaptive coping skills or personal traits. We found 18 resilience factors on a military unit (meso) level that are associated with the psychological resilience of soldiers (RQ2); most were labeled as the theme social environment, where they were categorized most frequently as leadership behavior. We discovered 20 resilience factors on a military community (macro) level that are linked with the psychological resilience of soldiers (RQ3); they were assigned most frequently to the theme training, with the majority grouped as psychological skill training.

Findings were classified according to the level at which they primarily operated. In cases where a factor could be assigned to more than one level, classification was based on its primary source and mechanism of influence. See Supplementary File C for examples of potentially ambiguous factor classifications and their underlying coding decision.

### 3.4 Quantitative studies

The capacity for resilience was operationalized the most frequently (57.1%) with different versions of the Connor–Davidson Resilience Scale (Connor and Davidson, 2003). All the measures are listed in the Supplementary File G. Their psychometric parameters were appropriate.

The various resilience factors were operationalized with multiple measures. Well-established questionnaires were used in 71.4% of the studies (Cai et al., 2017; Charbonneau, 2019; Reyes et al., 2020; Rice and Liu, 2016; Yurgil et al., 2024). Their psychometric parameters were also appropriate. Two papers used self-developed items (Adler et al., 2023; Valladares-Garrido et al., 2022). See the data extraction form for further information regarding the measures and methods of data analysis (Supplementary File G).

Most studies (*n* = 5) investigated micro-level resilience factors (RQ1). In total, they tested seven resilience factors, which we merged into the theme intrapersonal resources. With regard to RQ2, one paper examined two meso-level resilience factors, which we assigned to the theme social environment. Two studies investigated macro-level resilience factors (RQ3), which we categorized under the theme organizational framework. The quantitative results are listed in Table 3.

**TABLE 3:** Resilience factors from quantitative studies.

Resilience factors	Description	Measure	Direction	Significance	Effect size
**Micro level**					
Theme: intrapersonal resources					
Adaptive cognitive emotion regulation^2^	Strategies for positively focused cognitive emotion regulation measured	CERQC (Garnefski et al., 2002)	Positive	*p* < 0.01	*r* = 0.355 (strength); *r* = 0.340 (tenacity); *r* = 0.291 (optimism)
Baseline resilience^7^	Self-perception of psychological resilience at baseline	CD-RISC (Connor and Davidson, 2003)	Higher baseline resilience predicted higher post-deployment resilience.	*p* < 0.001	OR = 4.04 [3.15–5.18]
Mindfulness^3^	One’s degree of attention regarding events unfolding in the present	MAAS (Brown and Ryan, 2003)	Positive	*p* < 0.001	*r* = 0.37
Optimism^4^	Individual differences in generalized optimism versus pessimism	LOT-R (Scheier and Carver, 1985)	Positive	*p* < 0.001	β = 0.292 R^2^ = 0.082
Positive emotions^7^	Extent to which participants experience 10 positive emotions over the past few weeks	PANAS Schedule (Watson et al., 1988)	Higher positive emotions predicted higher post-deployment resilience	*p* < 0.001	OR = 4.04 [3.15–5.18]
Positive reframing^5^	Emotion-focused coping strategy	Brief COPE (Cooper et al., 2008)	Positive	*p* = 0.005	β = 0.275 Model R^2^ = 0.565^a^
Self-esteem^4^	Self-perception of global self-worth	RSES (Rosenberg, 1965)	Positive	*p* < 0.001	β = 0.486 R^2^ = 0.236
**Meso level**					
Theme: social environment					
Interpersonal support^7^	Self-perception of general interpersonal support	ISEL (Cohen and Hoberman, 1983)	Greater interpersonal support predicted higher post-deployment resilience	*p* < 0.001	OR = 4.12 [3.20–5.31]
Unit support^7^	Self-perception of support from military unit and leaders	DRRI (Vogt et al., 2013)	Greater unit support predicted higher post-deployment resilience	*p* = 0.001	OR = 1.52 [1.18–1.95]
**Macro level**					
Theme: organizational framework					
Wellness checks^1^	One-on-one mandatory and confidential annual counseling with mental health professionals, without medical context and records	Items (self-developed)	Positive	*p* = 0.03	B = 0.06 SE = 0.03
Months in assistance operation^6^	Number of months participants served in the COVID-19 assistance operation in the home country	Items (self-developed)	Positive	*p* = 0.021	PR = 1.35 [1.05–1.75]

Superscript number indicate which study reported the factor. The number refers to the study ID, which is provided in Table 1. CERQC, Cognitive Emotion Regulation Questionnaire–Chinese version; CD-RISC, Connor–Davidson Resilience Scale; MAAS, Mindful Attention Awareness Scale; LOT-R, Life Orientation Test – Revised; PANAS, Positive and Negative Affect; Brief COPE, Brief COPE Inventory; RSES, Rosenberg Self-Esteem Scale; ISEL, Interpersonal Support Evaluation List; DRRI, Deployment Risk and Resilience Inventory.

^a^R^2^ refers to the overall regression model.

## 4 Discussion

Our review yielded a few studies incorporating micro-, meso-, and macro-level factors that were linked with the capacity for resilience in the military. Both qualitative and quantitative studies most frequently reported micro-level factors, particularly personal traits or skills and adaptive coping skills. In the quantitative studies, we found an underrepresentation of meso- and macro-level factors. Moreover, no quantitative study dealt with multilevel resilience factors with nested data. In the following sections, we highlight our key findings and discuss the strengths and limitations of our study.

### 4.1 Structural and behavioral prevention

Our findings support other research (Dragonetti et al., 2020; Sinclair and Cheung, 2017; Steffens and Haslam, 2017) linking micro-level factors such as adaptive coping skills (e.g., positive reframing; Rice and Liu, 2016), personal traits or skills (e.g., optimism; Reyes et al., 2020), or knowledge (e.g., acquiring necessary skills and traits required for future operational tasks; Abraham et al., 2018), to resilience. Additionally, macro-level factors regarding the training of psychological (Doody et al., 2022) and job-related skills (Abraham et al., 2018) were identified. These results reinforce the practice of developing and implementing resilience training programs in the armed forces (Sun et al., 2024) and indicate that training in military and psychological skills may enhance resilience in soldiers. Military training prepares soldiers to accomplish operations successfully, while helping to identify those who are eligible for service (Nindl et al., 2018). Additionally, gaining field experience enhances soldiers’ readiness for future situations. Moreover, psychological skill training helps to improve their personal skills, enabling them to adaptively manage challenging situations (Crane et al., 2019; Zueger et al., 2023). Accordingly, training in these areas is important for prevention efforts.

However, psychological skill training emphasizes the importance of individual factors of resilience, placing the responsibility for developing resilience on the individual. The higher the level of stress an individual experiences, the more important other characteristics become. According to Ungar (2006), Ungar et al. (2023), resilience is both (a) an individual’s capacity to navigate toward resources in the face of adversity and (b) the provision of and experience with these resources. Although Ungar’s (2006) research focuses on resilience in children and adolescents, we consider his work as a valuable contribution to resilience in organizations. His understanding of resilience shifts the attention more toward the organization being responsible for implementing a resilient environment. Following Ungar (2011), we emphasize that the resources that individuals need to experience resilience should be made available and accessible by those who are in positions of power. To foster resilience, we thus recommend that organizations should first modify structural conditions.

Our review identified several macro-level resilience factors related hereto. For example, we found that recovery-related policies may improve soldiers’ resilience (Doody et al., 2022). This is consistent with earlier findings that mandatory dwell time supports outcomes of resilience (Adler and Saboe, 2017). Moreover, our review showed that organizational resources, such as low-threshold support services (e.g., wellness checks; Adler et al., 2023), the availability of and access to such services (e.g., availability of a counselor during a mission), or the provision of access to information (Doody et al., 2022) can aid in developing resilience among members of the military. Furthermore, other organizational conditions can help soldiers maintain their resilience; for example, soldiers’ resilience can be improved through measures that support the perceived equality between soldiers (e.g., no hierarchical differences in sleeping accommodations among soldiers; Doody et al., 2022). Thus, future research should consider the potential of structural conditions to establish a work environment that fosters resilience.

### 4.2 Social and cultural characteristics

In our review, we identified micro- and meso-level resilience factors related to social or cultural characteristics within the military. Social characteristics can be used to build an environment that promotes resilience because social resources shape the behavior of both individuals and organizations (Bergström and Dekker, 2014), especially in the military (Kanapeckaitë and Bagdžiūnienë, 2024; Polusny and Erbes, 2023). Moreover, social processes can play an important role in forming and sustaining an organizational culture that contributes to resilience as well (Adler and Saboe, 2017).

Resilience factors that describe the social dynamics between individuals were mainly classified as meso-level factors. More specifically, our review identified resilience factors that describe the social interaction among soldiers, their comrades, and commanders. Similarly, supportive relationships among these groups, in addition to social connectedness and support, directly and positively influence soldiers’ resilience (Abraham et al., 2018; Doody et al., 2022; Yurgil et al., 2024). Furthermore, the social environment can be related to soldiers’ resilience. For example, social characteristics can be used during informal training, when soldiers can share necessary knowledge and skills that are not considered in official training sessions (Doody et al., 2022). Additionally, social resources can become noticeable on the micro level, as well. For example, interpersonal resources (e.g., networking) or knowledge (e.g., knowledge and skills sharing) can help soldiers maintain a supportive network and contribute to resilience (Doody et al., 2022).

Our review supports recent findings showing that leadership plays a significant role in protecting soldiers from negative outcomes (Boga, 2023; Gomes et al., 2022). That is, how subordinates perceive their commander’s behavior promotes their capacity for resilience. Our results indicate that leadership styles are positively associated with resilience when they are characterized by, for example, commitment, integrity, role-modeling (Abraham et al., 2018), and transparency (Doody et al., 2022). Additionally, those in senior ranks influence the shared norms, values, and beliefs of soldiers with their behavior and may become role models who can reduce stigma-related barriers in the military.

This is important because the specific military culture is crucial when investigating resilience (Simmons and Yoder, 2013). Military culture encourages soldiers to develop a suitable military identity, which is characterized by beliefs, values, and attachment to the organization and which helps soldiers fulfill their duties. As our results indicate, knowledge about the military culture (Abraham et al., 2018) and the development of a military identity (expressing pride in the work; Doody et al., 2022) can contribute to resilience, as well as outcomes of resilience, as shown by other empirical evidence (Steffens et al., 2017). Thus, soldiers are expected to show physical and psychological strength under pressure, and resilience in the face of adversity (Boga, 2023; Britt et al., 2016a). Nevertheless, military culture may carry some risks that should be considered in resilience research. For example, not being resilient can become a source of stigma that may prevent individuals from seeking help when they need it (Kantor et al., 2017) because seeking treatment might lead to negative consequences (Adler, 2013).

According to Ungar (2006), individuals choose those resources that will most likely positively influence the outcomes of resilience, as determined by their culture and context. Ungar thereby draws attention to the crucial role a social group plays in giving meaning to provided resources. That is, resilience is influenced not only by soldiers’ own beliefs, values, and attachment but also by how their social environment functions and shapes their behavior (Turner and Oakes, 1986). Consequently, providing resources that promote the resilience of soldiers is needed, and these resources should be meaningful for soldiers; otherwise, they will not use them. We thus recommend that organizations not only provide the necessary resources but also draw attention to their cultural and contextual characteristics (e.g., measures of stigma reduction).

In line with Adler and Saboe (2017), we emphasize that the language an organization uses to discuss resilience is a key element in this regard. Using language that frames mental health impairments as occupational hazards (rather than individual weakness) is a crucial first step toward a military culture that promotes resilience. Additionally, leaders play a pivotal role, as their beliefs and values influence both the individuals and the groups they are commanding. Through their behavior, they can act as positive role models in reducing stigma (Britt et al., 2012). However, more research is needed in this regard, especially an in-depth exploration of those leadership behavioral patterns that reduce stigma-related concerns among soldiers.

## 5 Strengths and limitations

Our study is the first to systematically review micro-, meso-, and macro-level resilience factors within military populations by focusing on the capacity for resilience. This can be seen as the key strength of the study. Furthermore, by using a narrow definition of resilience, we considered other researchers’ criticisms of heterogeneous concepts of resilience. Moreover, we pre-registered our review, systematically searched the literature by using multiple databases and sources, and independently assessed the included studies’ quality and risk of bias.

However, the research has some limitations that should be considered when interpreting the findings. First, our review yielded only nine studies, seven quantitative and two qualitative, that met the eligibility criteria, resulting in a relatively small evidence base. Moreover, these studies varied considerably with regard to resilience measures and study design which limits their comparability. Second, to ensure quality, we limited the eligibility criteria to peer-reviewed journal articles published in English or German language. Consequently, relevant evidence reported in gray literature or other languages could be missed. Third, we did not include resilience factors beyond the military context (e.g., social support from non-work domains). Hence, we could have overlooked other relevant resilience factors from non-work domains. Fourth, the samples in the studies comprised mainly male participants, reflecting military demographics but limiting the transferability of findings to female military members. Fifth, most of the included studies were cross-sectional, limiting conclusions regarding potential causal relationships between micro-, meso-, and macro-level resilience factors and the capacity for resilience. Finally, record screening and full-text assessment were primarily conducted by one reviewer. Although cases of uncertainty were discussed with the co-authors until consensus was reached, the possibility of selection bias cannot be entirely excluded.

## 6 Conclusion and future directions

In our review, we determined that factors related to the capacity for resilience–particularly meso- and macro-level factors and multilevel factors with nested data–are still under-researched within the military. Given the high complexity and dynamics of resilience, multilevel investigations are needed to capture mechanisms such as individual resources, social dynamics, or structural conditions and to better understand their interactions (Polusny and Erbes, 2023). Moreover, a multilevel framework can examine how social interactions shape individual and organizational behavior (Bergström and Dekker, 2014) in military but also comparable occupations that face high-risk stressors such as first responders (e.g., firefighters, police officers, emergency health workers).

In terms of theoretical implications, future research should consider a multilevel approach to better understand the process of resilience building in high-risk occupations and help in developing more targeted interventions. In terms of practical implications, our synthesis provides insights that shift the responsibility for building a resilient environment from the individual to the organization. Therefore, organizations should modify organizational policies (e.g., recovery-related policies) and resources (e.g., provision of access to information) first. Moreover, they should develop measures to enhance organizational culture by improving the social dynamics and reducing stigma. In this regard, leaders can act as key agents because they serve as role models for their subordinates.

In conclusion, our synthesis facilitates a deeper understanding of what is meant by a resilient organization and how developing resilience can be balanced with other organizational responsibilities.

## Data Availability

The original contributions presented in this study are included in this article/Supplementary material, further inquiries can be directed to the corresponding author.
