# Supplementary material for: Soldiers’ resilience? A systematic review of micro-, meso-, and macro-level resilience factors in the military
Source: Front Psychol. 2026 Jul 22;17:1857328. doi: 10.3389/fpsyg.2026.1857328 (PMC13437443; doi:10.3389/fpsyg.2026.1857328)
Supplement: Supplementary file 1 [file Table_1.docx]

**Supplementary File A – Full Search Strategy**

**Full search strategy**

( resilien* not resilience training ) AND ( (military or military personnel or army or army personnel or armed forces or soldier* or deploy* or draftee*) not veteran* not service leaver* ) AND ( factor* or model* or dimension* or predict* or level* or promot* or faciliat* or enabl* )

**PsycINFO [Abstract]**

AB ( ( resilien* not resilience training ) AND ( (military or military personnel or army or army personnel or armed forces or soldier* or deploy* or draftee*) not veteran* not service leaver* ) AND ( factor* or model* or dimension* or predict* or level* or promot* or faciliat* or enabl* ) ) AND ( ZL "English" OR "German" )

| Search conducted: | 29/04/2022; filters applied: publication year 2000-2022; results: 798 |
| --- | --- |
| Last search conducted: | 31/05/2025; filters applied: publication year 04/2022-2025; results: 147 |

**Pubmed [Title, Abstract]**

(resilien*[Title/Abstract]) AND ((military[Title/Abstract]) OR ("military personnel"[Title/Abstract]) OR (army[Title/Abstract]) OR ("army personnel"[Title/Abstract]) OR ("armed forces"[Title/Abstract]) OR (soldier*[Title/Abstract]) OR (deploy*[Title/Abstract]) OR (draftee*[Title/Abstract])) AND ((factor*[Title/Abstract]) OR (model*[Title/Abstract]) OR (dimension*[Title/Abstract]) OR (predict*[Title/Abstract]) OR (level*[Title/Abstract]) OR (promot*[Title/Abstract]) OR (faciliat*[Title/Abstract]) OR (enabl*[Title/Abstract])) NOT ("resilience training"[Title/Abstract]) NOT (veteran [Title/Abstract]) NOT ("service leaver*"[Title/Abstract])

| Search conducted: | 29/04/2022; filters applied: English, German, from 2000 - current; results: 958 |
| --- | --- |
| Last search conducted: | Last search conducted: 31/05/2025; filters applied: English or German; from 2022-current; results: 695 |

**Scopus [Title, Abstract, Keywords]**

TITLE-ABS-KEY ( ( resilien* AND (military OR {military personnel} OR army OR {army personnel} OR {armed forces} OR soldier* OR deploy* OR draftee*) AND (factor* OR model* OR dimension* OR predict* OR level* OR promot* OR faciliat* OR enabl*) ) ) AND PUBYEAR > 1999 AND NOT {resilience training} AND NOT veteran* AND NOT {service leaver*} AND ( LIMIT-TO ( LANGUAGE,"English" ) OR LIMIT-TO ( LANGUAGE,"German" ) ) AND ( EXCLUDE ( SUBJAREA,"COMP" ) OR EXCLUDE ( SUBJAREA,"ENGI" ) OR EXCLUDE ( SUBJAREA,"MATH" ) OR EXCLUDE ( SUBJAREA,"ENVI" ) OR EXCLUDE ( SUBJAREA,"ENER" ) OR EXCLUDE ( SUBJAREA,"PHYS" ) OR EXCLUDE ( SUBJAREA,"MATE" ) OR EXCLUDE ( SUBJAREA,"EART" ) OR EXCLUDE ( SUBJAREA,"CHEM" ) OR EXCLUDE ( SUBJAREA,"CENG" ) )

| Search conducted: | 28/04/2022; results: 1295 |
| --- | --- |
| Last search conducted: | Last search conducted: 31/05/2025; filters applied: PUBYEAR > 2022; results: 607 |

*The preliminary searches in Scopus revealed that a substantial number of retrieved records fell outside the scope of this review (e.g., publications pertaining to IT security). To address this issue, we utilized Scopus's filtering capabilities, specifically the "Subject Areas" feature, which allows for the refinement of search results based on scientific disciplines. During the preliminary phase, we systematically screened the titles and abstracts of search results within each subject area to assess their relevance. Only those subject areas deemed pertinent to our research objectives were included in the main searches.*

**Web of Science [Abstract]**

(AB=(resilien*)) AND (AB=(military or military personnel or army or army personnel or armed forces or soldier* or deploy* or draftee*)) AND (AB=(factor* or model* or dimension* or predict* or level* or promot* or faciliat* or enabl*)) AND (LA==("GERMAN" OR "ENGLISH")) NOT (AB=(resilience training)) NOT (AB=( veteran*)) NOT (AB=( service leaver*)) and Psychiatry or Psychology Clinical or Public Environmental Occupational Health or Medicine General Internal or Psychology Multidisciplinary or Management or Family Studies or Neurosciences or Psychology or Clinical Neurology or Social Work or Multidisciplinary Sciences or Social Sciences Interdisciplinary or Political Science or Psychology Developmental or Nursing or Education Educational Research or Sociology or Business or Psychology Social or Psychology Applied or Behavioral Sciences or Sport Sciences or Biochemistry Molecular Biology or Genetics Heredity or Health Care Sciences Services or Social Sciences Biomedical or Health Policy Services or Criminology Penology or Endocrinology Metabolism or Operations Research Management Science or Communication or Psychology Experimental or Rehabilitation or Biology or Microbiology or Pharmacology Pharmacy or Religion or Substance Abuse or Critical Care Medicine or Hospitality Leisure Sport Tourism or Psychology Biological or Food Science Technology or Nutrition Dietetics or Emergency Medicine or Humanities Multidisciplinary or Integrative Complementary Medicine or Medicine Research Experimental or Physiology or Women S Studies or Dermatology or Education Scientific Disciplines or Literature or Obstetrics Gynecology or Cardiac Cardiovascular Systems or Cell Biology or Demography or Ergonomics or Industrial Relations Labor or Psychology Educational or Social Issues or Social Sciences Mathematical Methods or Transportation or Orthopedics (Web of Science Categories)

| Search conducted: | 30/04/2022; filters applied: Timespan: 2000-01-01 to 2022-04-30; results: 520 |
| --- | --- |
| Last search conducted: | 31/05/2025; filters applied: Timespan: 2022-04-01 – 2025-05-31; results: 67 |

*Our preliminary searches in Web of Science revealed that many retrieved records were outside the intended scope of this review (for instance, studies on IT security). To mitigate this, we made use of Web of Science’s built‑in filtering feature—Web of Science Categories—which allows results to be refined by scientific discipline. In the preliminary phase, we systematically screened the titles and abstracts within each category to assess relevance. Only categories deemed directly pertinent to our research objectives were included in subsequent, more targeted searches.*
