# Supplementary material for: Soldiers’ resilience? A systematic review of micro-, meso-, and macro-level resilience factors in the military
Source: Front Psychol. 2026 Jul 22;17:1857328. doi: 10.3389/fpsyg.2026.1857328 (PMC13437443; doi:10.3389/fpsyg.2026.1857328)
Supplement: Supplementary file 3 [file Table_3.docx]

**Supplementary File F – Characteristics of the included studies**

**Table 1. Characteristics of the included studies.**

| # | Study | Country | Type | Design | Measure |
| --- | --- | --- | --- | --- | --- |
| ^1^ | Adler et al. (2023) | USA | Quan. | Cross-sectional | BRS |
| ^2^ | Cai et al. (2017) | CHN | Quan. | Cross-sectional | CD-RISC |
| ^3^ | Charbonneau (2019) | CAN | Quan. | Cross-sectional | BRS |
| ^4^ | Reyes et al. (2020) | PHL | Quan. | Cross-sectional | CD-RISC |
| ^5^ | Rice and Liu (2016) | USA | Quan. | Cross-sectional | RS-14 |
| ^6^ | Valladares-Garrido et al. (2022) | PER | Quan. | Cross-sectional | CD-RISC |
| ^7^ | Yurgil et al. (2024) | USA | Quan | Longitudinal | CD-RISC |
| ^8^ | Abraham et al. (2018) | USA | Qual. | Cross-sectional | Semi-structured interviews |
| ^9^ | Doody et al. (2022) | IRL | Qual. | Cross-sectional | Semi-structured interviews |

USA = United States of America; CHN = China; CAN = Canada; PHL = Philippines; PER = Peru; IRL = Ireland; Quan. = quantitative; Qual. = qualitative; BRS = Brief Resilience Scale (Smith et al., 2008); CD-RISC = Connor-Davidson Resilience Scale (Connor and Davidson, 2003); RS-14 = 14-Item Resilience Scale (Wagnild, 2011, as cited in Rice and Liu., 2016).
