# Supplementary material for: Soldiers’ resilience? A systematic review of micro-, meso-, and macro-level resilience factors in the military
Source: Front Psychol. 2026 Jul 22;17:1857328. doi: 10.3389/fpsyg.2026.1857328 (PMC13437443; doi:10.3389/fpsyg.2026.1857328)
Supplement: Supplementary file 4 [file Table_4.docx]

**Supplementary File E – Excluded records**

**Table E1. Excluded records after full text screening grouped by exclusion criteria**

| **I. No focus on capacity of resilience (n = 36)** | |
| --- | --- |
| 1 | Adler, A. B., Bliese, P. D., Barsade, S. G., & Sowden, W. J. (2022). Hitting the mark: The influence of emotional culture on resilient performance. *The Journal of Applied Psychology*, *107*(2), 319–327. <https://doi.org/10.1037/apl0000897> |
| 2 | Bennett, N., Lawrence-Wood, E., & McFarlane, A. (2024). Identifying psychological risk and resilience in high-risk military personnel. *BMJ Military Healtt*, 170(5), 390–395. <https://doi.org/10.1136/military-2024-002724> |
| 3 | Bonanno, G. A., Mancini, A. D., Horton, J. L., Powell, T. M., Leardmann, C. A., Boyko, E. J., Wells, T. S., Hooper, T. I., Gackstetter, G. D., & Smith, T. C. (2012). Trajectories of trauma symptoms and resilience in deployed U.S. military service members: Prospective cohort study. *The British Journal of Psychiatry: The Journal of Mental Science*, *200*(4), 317–323. <https://doi.org/10.1192/bjp.bp.111.096552> |
| 4 | Call, D., Pitcock, J., & Pyne, J. (2015). Longitudinal evaluation of the relationship between mindfulness, general distress, anxiety, and PTSD in a recently deployed National Guard sample. *Mindfulness*, *6*(6), 1303–1312. psyh. <https://doi.org/10.1007/s12671-015-0400-0> |
| 5 | Carter-Visscher, R., Polusny, M. A., Murdoch, M., Thuras, P., Erbes, C. R., & Kehle, S. M. (2010). Predeployment gender differences in stressors and mental health among U.S. National Guard troops poised for Operation Iraqi Freedom deployment. *Journal of Traumatic Stress, 23*(1), 78–85. <https://doi.org/10.1002/jts.20481> |
| 6 | Caska, C. M., & Renshaw, K. D. (2013). Personality traits as moderators of the associations between deployment experiences and PTSD symptoms in OEF/OIF service members. *Anxiety, Stress, and Coping, 26*(1), 36–51. <https://doi.org/10.1080/10615806.2011.638053> |
| 7 | Chopik, W., Kelley, W., Vie, L., Lester, P., Bonett, D., Lucas, R., & Seligman, M. (2022). Individual and experiential predictors of character development across the deployment cycle. *European Journal of Personality*. <https://doi.org/10.1177/08902070211012931> |
| 8 | Dell, L., Casetta, C., Benassi, H., Cowlishaw, S., Agathos, J., O’Donnell, M., Crane, M., Lewis, V., Pacella, B., Terhaag, S., Morton, D., McFarlane, A., Bryant, R., & Forbes, D. (2023). Mental health across the early years in the military. *Psychological Medicine, 53*(8), 3683–3691. <https://doi.org/10.1017/S0033291722000332> |
| 9 | Eid, J., & Johnsen, B. H. (2002). Acute stress reactions after submarine accidents. *Military Medicine, 167*(5), 427–431. Scopus. <https://doi.org/10.1093/miled.167.5.427> |
| 10 | Gorman, L. A., Huebner, A. J., Hirschfeld, M. K., Sankar, S., Blow, A. J., Guty, D., Kees, M., & Ketner, J. S. (2016). A Comparative Case Study of Risk, Resiliency, and Coping Among Injured National Guard. *Military Medicine, 181*(5 Suppl), 70–76. <https://doi.org/10.7205/MILMED-D-15-00126> |
| 11 | Gottschall, S., & Guérin, E. (2021). Organizational and non-organizational risk and resilience factors associated with mental health and well-being in the royal canadian navy. *Current Psychology: A Journal for Diverse Perspectives on Diverse Psychological Issues*. psyh. <https://doi.org/10.1007/s12144-021-01508-x> |
| 12 | Hickling, E. J., Gibbons, S., Barnett, S. D., & Watts, D. (2011). The psychological impact of deployment on OEF/OIF healthcare providers. *Journal of Traumatic Stress, 24*(6), 726–734. <https://doi.org/10.1002/jts.20703> |
| 13 | Highland, K. B., Costanzo, M., Jovanovic, T., Norrholm, S. D., Ndiongue, R., Reinhardt, B., Rothbaum, B., & Roy, M. J. (2015). Biomarkers of post-deployment resilience among military service members. *Neurobiology of Stress, 2*, 62–66. <https://doi.org/10.1016/j.ynstr.2015.07.001> |
| 14 | Hoopsick, R. A., Homish, D. L., Collins, R. L., Nochajski, T. H., Read, J. P., Bartone, P. T., & Homish, G. G. (2021). Resilience to mental health problems and the role of deployment status among U.S. Army Reserve and National Guard Soldiers. *Social Psychiatry and Psychiatric Epidemiology, 56*(7), 1299–1310. <https://doi.org/10.1007/s00127-020-01899-5> |
| 15 | Hoopsick, R. A., Homish, D. L., Collins, R. L., Nochajski, T. H., Read, J. P., & Homish, G. G. (2020). Is deployment status the critical determinant of psychosocial problems among reserve/guard soldiers? *Psychological Services, 17*(4), 461–471. <https://doi.org/10.1037/ser0000331> |
| 16 | Hoopsick, R. A., Homish, D. L., Vest, B. M., Bartone, P. T., & Homish, G. G. (2021). Resilience to Hazardous Drinking Among Never-Deployed Male United States Army Reserve and National Guard Soldiers. *Alcoholism, Clinical and Experimental Research, 45*(3), 566–576. <https://doi.org/10.1111/acer.14561> |
| 17 | Kessler, R. C., Bossarte, R. M., Hwang, I., Luedtke, A., Naifeh, J. A., Nock, M. K., Petukhova, M., Sadikova, E., Sampson, N. A., Sverdrup, E., Zubizarreta, J. R., Wager, S., Wagner, J., Stein, M. B., & Ursano, R. J. (2024). A prediction model for differential resilience to the effects of combat-related stressors in US army soldiers. *International Journal of Methods in Psychiatric Research*, 33(4), e70006. <https://doi.org/10.1002/mpr.70006> |
| 18 | Kline, A., Ciccone, D. S., Weiner, M., Interian, A., St Hill, L., Falca-Dodson, M., Black, C. M., & Losonczy, M. (2013). Gender differences in the risk and protective factors associated with PTSD: a prospective study of National Guard troops deployed to Iraq. *Psychiatry, 76*(3), 256–272. <https://doi.org/10.1521/psyc.2013.76.3.256> |
| 19 | Luning, C., Attoh, P., Gong, T., & Fox, J. (2022). A Culture of Organizational Grit From the Perspective of US Military Officers: A Qualitative Inquiry. *Armed Forces & Society, 48*(3), 542–570. <https://doi.org/10.1177/0095327X20985197> |
| 20 | Maguen, S., Turcotte, D. M., Peterson, A. L., Dremsa, T. L., Garb, H. N., McNally, R. J., & Litz, B. T. (2008). Description of risk and resilience factors among military medical personnel before deployment to Iraq. *Military Medicine, 173*(1), 1–9. <https://doi.org/10.7205/milmed.173.1.1> |
| 21 | McLarnon, M. J. W., Rothstein, M. G., & King, G. A. (2021). Resiliency to adversity in military personnel: The role of self-regulation. *Military Psychology, 33*(2), 104–114. psyh. <https://doi.org/10.1080/08995605.2021.1897492> |
| 22 | Melkevik, O., Jeppesen, L. S., Folke, S., & Nielsen, A. B. S. (2025). Low neuroticism as an indicator of resilience: A longitudinal study of Danish soldiers before, during and after deployment. *European Journal of Psychotraumatology*, 16(1), 2476810. <https://doi.org/10.1080/20008066.2025.2476810> |
| 23 | Minassian, A., Geyer, M. A., Baker, D. G., Nievergelt, C. M., O’Connor, D. T., & Risbrough, V. B. (2014). Heart rate variability characteristics in a large group of active-duty marines and relationship to posttraumatic stress. *Psychosomatic Medicine, 76*(4), 292–301. <https://doi.org/10.1097/PSY.0000000000000056> |
| 24 | Nasky, K. M., Hines, N. N., & Simmer, E. (2009). The USS Cole bombing: Analysis of pre-existing factors as predictors for development of post-traumatic stress or depressive disorders. *Military Medicine, 174*(7), 689–694. <https://doi.org/10.7205/milmed-d-04-3608> |
| 25 | Olson, J. R., Welsh, J. A., Perkins, D. F., & Ormsby, L. (2018). Individual, family, and community predictors of PTSD symptoms following military deployment. *Family Relations: An Interdisciplinary Journal of Applied Family Studies, 67*(5), 615–629. psyh. <https://doi.org/10.1111/fare.12343> |
| 26 | Polusny, M. A., Erbes, C. R., Murdoch, M., Arbisi, P. A., Thuras, P., & Rath, M. B. (2011). Prospective risk factors for new-onset post-traumatic stress disorder in National Guard soldiers deployed to Iraq. *Psychological Medicine, 41*(4), 687–698. <https://doi.org/10.1017/S0033291710002047> |
| 27 | Polusny, M. A., Marquardt, C. A., Campbell, E. H., Filetti, C. R., Noël, V. V., Disner, S. G., Schaefer, J. D., Davenport, N., Lissek, S., Noorbaloochi, S., Sponheim, S. R., & Erbes, C. R. (2021). Advancing Research on Mechanisms of Resilience (ARMOR) Longitudinal Cohort Study of New Military Recruits: Results from a Feasibility Pilot Study. *Research in Human Development, 18*(3), 212–229. <https://doi.org/10.1080/15427609.2021.1964898> |
| 28 | Reijnen, A., Geuze, E., & Vermetten, E. (2017). Individual variation in plasma oxytocin and vasopressin levels in relation to the development of combat-related PTSD in a large military cohort. *Journal of Psychiatric Research, 94*, 88–95. <https://doi.org/10.1016/j.jpsychires.2017.06.010> |
| 29 | Riolli, L., Savicki, V., & Spain, E. (2010). Positive emotions in traumatic conditions: Mediation of appraisal and mood for military personnel. *Military Psychology, 22*(2), 207–223. psyh. <https://doi.org/10.1080/08995601003638975> |
| 30 | Schäfer, J., Wittchen, H.-U., Höfler, M., Heinrich, A., Zimmermann, P., Siegel, S., & Schönfeld, S. (2015). Is trait resilience characterized by specific patterns of attentional bias to emotional stimuli and attentional control? *Journal of Behavior Therapy and Experimental Psychiatry, 48*, 133–139. <https://doi.org/10.1016/j.jbtep.2015.03.010> |
| 31 | Seelig, A. D., Jacobson, I. G., Donoho, C. J., Trone, D. W., Crum-Cianflone, N. F., & Balkin, T. J. (2016). Sleep and Health Resilience Metrics in a Large Military Cohort. *Sleep, 39*(5), 1111–1120. <https://doi.org/10.5665/sleep.5766> |
| 32 | Shen, Y.-C., Arkes, J., & Lester, P. B. (2017). Association between baseline psychological attributes and mental health outcomes after soldiers returned from deployment. *BMC Psychology, 5*(1), 32. <https://doi.org/10.1186/s40359-017-0201-4> |
| 33 | Sudom, K. A., Lee, J. E. C., & Zamorski, M. A. (2014). A longitudinal pilot study of resilience in Canadian military personnel. Stress and Health : *Journal of the International Society for the Investigation of Stress, 30*(5), 377–385. <https://doi.org/10.1002/smi.2614> |
| 34 | Vest, B. M., Heavey, S. C., Homish, D. L., & Homish, G. G. (2017). Marital Satisfaction, Family Support, and Pre-Deployment Resiliency Factors Related to Mental Health Outcomes for Reserve and National Guard Soldiers. *Military Behavioral Health, 5*(4), 313–323. <https://doi.org/10.1080/21635781.2017.1343694> |
| 35 | Ward, R. N., Carlson, K. J., Erickson, A. J., Yalch, M. M., & Brown, L. M. (2021). Associations of humor, morale, and unit cohesion on posttraumatic stress disorder symptoms. *Military Psychology*. psyh. <https://doi.org/10.1080/08995605.2021.1996103> |
| 36 | Webb-Murphy, J. A., De La Rosa, G. M., Schmitz, K. J., Vishnyak, E. J., Raducha, S. C., Roesch, S. C., & Johnston, S. L. (2015). Operational Stress and Correlates of Mental Health Among Joint Task Force Guantanamo Bay Military Personnel. *Journal of Traumatic Stress, 28*(6), 499–504. <https://doi.org/10.1002/jts.22057> |
| **II. No focus on resilience factor(s) on at least one level (n = 32)** | |
| 1 | Afek, A., Ben-Avraham, R., Davidov, A., Berezin Cohen, N., Ben Yehuda, A., Gilboa, Y., & Nahum, M. (2020). Psychological Resilience, Mental Health, and Inhibitory Control Among Youth and Young Adults Under Stress. *Frontiers in Psychiatry, 11*, 608588. <https://doi.org/10.3389/fpsyt.2020.608588> |
| 2 | Britt, T. W., Adler, A. B., & Fynes, J. (2021). Perceived resilience and social connection as predictors of adjustment following occupational adversity. *Journal of Occupational Health Psychology, 26*(4), 339–349. <https://doi.org/10.1037/ocp0000286> |
| 3 | Cabrera, O. A., Trachik, B. J., Ganulin, M. L., Dretsch, M. N., & Adler, A. B. (2022). Factor structure and measurement invariance of the brief resilience scale in deployed and non-deployed soldiers. *Occupational Health Science*. psyh. <https://doi.org/10.1007/s41542-022-00138-4> |
| 4 | Cabrera, O. A., Trachik, B. J., Ganulin, M. L., Dretsch, M. N., & Adler, A. B. (2023). Longitudinal measurement invariance and growth curve modeling of psychological resilience across the deployment cycle. *Military Psychology*. psyh. <https://doi.org/10.1080/08995605.2023.2188846> |
| 5 | Cafferky, B., Norton, A., & Travis, W. J. (2017). Air Force Chaplains’ Perceived Effectiveness on Service Member’s Resilience and Satisfaction. *Journal of Health Care Chaplaincy, 23*(2), 45–66. <https://doi.org/10.1080/08854726.2016.1250521> |
| 6 | Campbell-Sills, L., Kessler, R. C., Ursano, R. J., Sun, X., Taylor, C. T., Heeringa, S. G., Nock, M. K., Sampson, N. A., Jain, S., & Stein, M. B. (2018). Predictive validity and correlates of self-assessed resilience among U.S. Army soldiers. *Depression and Anxiety, 35*(2), 122–131. <https://doi.org/10.1002/da.22694> |
| 7 | Chang, K., & Taormina, R. J. (2011). Reduced secondary trauma among Chinese earthquake rescuers: A test of correlates and life indicators. *Journal of Loss and Trauma, 16*(6), 542–562. psyh. <https://doi.org/10.1080/15325024.2011.600682> |
| 8 | Choi, K. W., Chen, C.-Y., Ursano, R. J., Sun, X., Jain, S., Kessler, R. C., Koenen, K. C., Wang, M.-J., Wynn, G. H., Campbell-Sills, L., Stein, M. B., & Smoller, J. W. (2020). Prospective study of polygenic risk, protective factors, and incident depression following combat deployment in US Army soldiers. *Psychological Medicine, 50*(5), 737–745. <https://doi.org/10.1017/S0033291719000527> |
| 9 | Foster, E. M. (2011). Deployment and the citizen soldier: Need and resilience. *Medical Care, 49*(3), 301–312. <https://doi.org/10.1097/MLR.0b013e318202abfc> |
| 10 | Georgoulas-Sherry, V., & Kelly, D. R. (2019). Resilience, grit, and hardiness: Determining the relationships amongst these constructs through structural equation modeling techniques. *Journal of Positive Psychology and Wellbeing, 3*(2), 165–178. Scopus. |
| 11 | Hoopsick, R. A., Benson, K. R., Homish, D. L., & Homish, G. G. (2019). Resiliency factors that protect against post-deployment drug use among male US Army Reserve and National Guard soldiers. *Drug and Alcohol Dependence, 199*, 42–49. <https://doi.org/10.1016/j.drugalcdep.2019.02.017> |
| 12 | Iversen, Z. A., Almagor Tikotzki, M., & Kurman, J. (2023). Psychological resilience: Predictors and measurement among Israel Defense Force combat officer candidates. *Military Psychology, 35*(6), 493–506. <https://doi.org/10.1080/08995605.2022.2127986> |
| 13 | Jensen, T., & Bowen, G. (2018). Informing efforts to prevent family maltreatment among airmen: A focus on personal resilience. *Journal of Family Social Work, 21*(2), 129–151. <https://doi.org/10.1080/10522158.2017.1410270> |
| 14 | Layman, P. G., Sanford, K., Myers, D. R., Dolan, S., Ellor, J. W., Morissette, S. B., Whitacre, J., & Crow, J. (2019). Intimate partner cohesion and military unit cohesion: Different types of interpersonal relationships each uniquely predict soldier well-being. *Military Psychology, 31*(3), 178–186. psyh. <https://doi.org/10.1080/08995605.2019.1579606> |
| 15 | Ledford, A. K., Dixon, D., Luning, C. R., Martin, B. J., Miles, P. C., Beckner, M., Bennett, D., Conley, J., & Nindl, B. C. (2020). Psychological and Physiological Predictors of Resilience in Navy SEAL Training. *Behavioral Medicine, 46*(3–4), 290–301. <https://doi.org/10.1080/08964289.2020.1712648> |
| 16 | Ligeza, N., Larson, A., & DeBeliso, M. (2022). Resilience, Psychological Stress, Physical Activity, and BMI among United States Air National Guardsmen: The COVID-19 Pandemic. *Journal of Lifestyle Medicine, 12*(1), 26–36. <https://doi.org/10.15280/jlm.2022.12.1.26> |
| 17 | Mitchell, N. A., McCauley, M., O’Brien, D., & Wilson, C. E. (2023). Mental health and resilience in the Irish defense forces during the COVID-19 global pandemic. *Military Psychology, 35*(5), 383–393. psyh. <https://doi.org/10.1080/08995605.2021.2007728> |
| 18 | Moore, T. M., Risbrough, V. B., Baker, D. G., Larson, G. E., Glenn, D. E., Nievergelt, C. M., Maihofer, A., Port, A. M., Jackson, C. T., Ruparel, K., & Gur, R. C. (2017). Effects of military service and deployment on clinical symptomatology: The role of trauma exposure and social support. *Journal of Psychiatric Research, 95*, 121–128. <https://doi.org/10.1016/j.jpsychires.2017.08.013> |
| 19 | Mourtakos, S., Vassiliou, G., Papageorgiou, C., Kontoangelos, K., Philippou, A., Bersimis, F., Geladas, N., Koutsilieris, M., Sidossis, L. S., Tsirmpas, C., & Papageorgiou, C. (2021). Resilience of the Hellenic navy seals assessed by heart rate variability during cognitive tasks. *Physiology & Behavior, 239*, 113437. <https://doi.org/10.1016/j.physbeh.2021.113437> |
| 20 | Nahum, M., Sinvani, R.-T., Afek, A., Ben Avraham, R., Jordan, J. T., Ben Shachar, M. S., Ben Yehuda, A., Berezin Cohen, N., Davidov, A., & Gilboa, Y. (2023). Inhibitory control and mood in relation to psychological resilience: An ecological momentary assessment study. *Scientific Reports, 13*(1), 13151. <https://doi.org/10.1038/s41598-023-40242-1> |
| 21 | Prontenko, K. V., Okhrimenko, I. М., Yevdokimova, O. O., Mannapova, K. R., Filonenko, V. M., Tverdokhvalova, I. L., & Bondarenko, L. O. (2023). Peculiarities of formation of cadets’ psychological resilience and physical readiness for combat stress. *Wiadomosci Lekarskie*, *76*(6), 1450–1456. <https://doi.org/10.36740/WLek202306118> |
| 22 | Qiu, R., Gong, Y., Cao, Y., You, X., & Zhu, X. (2023). Influence of self-efficacy on male military pilots’ capability to handle special situations: A moderated mediation model. *Scientific Reports, 13*(1), 10794. <https://doi.org/10.1038/s41598-023-38009-9> |
| 23 | Shi, L., Xin, S., Li, D.-N., Song, P.-Y., Tian, Y., Ren, F., Huan, J.-L., & Wang, Y. (2025). The mediating role of resilience in job satisfaction and burnout among Chinese plateau military personnel. *Scientific Reports*, 15(1), 13785. <https://doi.org/10.1038/s41598-025-97673-1> |
| 24 | Sohail, M., & Ahmad, G. (2021). Resilience, Psychological Well-being, and Emotional Regulation: A Comparative Study of Military Personnel Vs. Civilian Population. *Pakistan Journal of Psychological Research, 36*(1), 37–49. Scopus. <https://doi.org/10.33824/PJPR.2021.36.1.03> |
| 25 | Szabó, Z. P., Kun, Á., Balogh, B. E., Simon, E., & Csike, T. (2022). Dark and Strong?! The associations between dark personality traits, mental toughness and resilience in Hungarian student, employee, leader, and military samples. *Personality and Individual Differences, 186*. Scopus. <https://doi.org/10.1016/j.paid.2021.111339> |
| 26 | Tavares, J. P., Vieira, L. S., Pai, D. D., Souza, S. B. C. de, Ceccon, R. F., & Machado, W. de L. (2021). Network of correlations between quality of life, resilience and effort-reward imbalance in military police officers. *Ciencia & Saude Coletiva, 26*(5), 1931–1940. <https://doi.org/10.1590/1413-81232021265.10702019> |
| 27 | Taylor, M. K., Rolo, C., Stump, J., Mayo, J., Hernandez, L. M., & Gould, D. R. (2019). Psychological Strategies During Military Training Are Linked to Resilience in US Navy Explosive Ordnance Disposal Operators. *Journal of Special Operations Medicine, 19*(1), 61–65. |
| 28 | Valor-Segura, I., Navarro-Carrillo, G., Extremera, N., Lozano, L. M., García-Guiu, C., Roldán-Bravo, M. I., & Ruiz-Moreno, A. (2020). Predicting Job Satisfaction in Military Organizations: Unpacking the Relationship Between Emotional Intelligence, Teamwork Communication, and Job Attitudes in  Spanish Military Cadets. *Frontiers in Psychology, 11*, 875. <https://doi.org/10.3389/fpsyg.2020.00875> |
| 29 | Van Breda, A. D. (2008). The military social health index: A partial multicultural validation. *Military Medicine, 173*(5), 480–487. <https://doi.org/10.7205/milmed.173.5.480> |
| 30 | Whealin, J. M., Nelson, D., Stotzer, R., Guerrero, A., Carpenter, M., & Pietrzak, R. H. (2015). Risk and resilience factors associated with posttraumatic stress in ethno-racially diverse National Guard members in Hawai׳i. *Psychiatry Research, 227*(2–3), 270–277. <https://doi.org/10.1016/j.psychres.2015.02.023> |
| 31 | Wooten, N. R. (2012). Deployment cycle stressors and post-traumatic stress symptoms in Army National Guard women: The mediating effect of resilience. *Social Work in Health Care, 51*(9), 828–849. <https://doi.org/10.1080/00981389.2012.692353> |
| 32 | Zimmermann, P., Firnkes, S., Kowalski, J., Backus, J., Alliger-Horn, C., Willmund, G., Hellenthal, A., Bauer, A., Petermann, F., & Maercker, A. (2015). [Mental disorders in German soldiers after deployment—Impact of personal values and resilience]. *Psychiatrische Praxis, 42*(8), 436–442. <https://doi.org/10.1055/s-0034-1370242> |
| **III. No quantitative, qualitative or mixed methods used (n = 25)** | |
| 1 | Zou, M., Liu, B., Ji, J., Ren, L., Wang, X., & Li, F. (2024). The Relationship Between Negative Coping Styles, Psychological Resilience, and Positive Coping Styles in Military Personnel: A Cross-Lagged Analysis. *Psychology Research and Behavior Management, 17*, 13–21. <https://doi.org/10.2147/PRBM.S447096> |
| 2 | Bartone, P. T. (2006). Resilience under military operational stress: Can leaders influence hardiness? *Military Psychology, 18*(Suppl), S131–S148. psyh. <https://doi.org/10.1207/s15327876mp1803s_10> |
| 3 | Bates, M. J., Bowles, S., Hammermeister, J., Stokes, C., Pinder, E., Moore, M., Fritts, M., Vythilingam, M., Yosick, T., Rhodes, J., Myatt, C., Westphal, R., Fautua, D., Hammer, P., & Burbelo, G. (2010). Psychological fitness. *Military Medicine, 175*(Suppl 1), 21–38. psyh. <https://doi.org/10.7205/MILMED-D-10-00073> |
| 4 | Beckner, M. E., Main, L., Tait, J. L., Martin, B. J., Conkright, W. R., & Nindl, B. C. (2022). Circulating biomarkers associated with performance and resilience during military operational stress. *European Journal of Sport Science, 22*(1), 72–86. <https://doi.org/10.1080/17461391.2021.1962983> |
| 5 | Bowen, G. L., & Martin, J. A. (2011). The resiliency model of role performance for service members, veterans, and their families: A focus on social connections and individual assets. *Journal of Human Behavior in the Social Environment, 21*(2), 162–178. psyh. <https://doi.org/10.1080/10911359.2011.546198> |
| 6 | Bowles, S. V., & Bates, M. J. (2010). Military organizations and programs contributing to resilience building. *Military Medicine, 175*(6), 382–385. psyh. <https://doi.org/10.7205/MILMED-D-10-00099> |
| 7 | Cacioppo, J. T., Reis, H. T., & Zautra, A. J. (2011). Social resilience: The value of social fitness with an application to the military. *The American Psychologist, 66*(1), 43–51. <https://doi.org/10.1037/a0021419> |
| 8 | Caravalho, J. J. (2015). Improving Soldier Health and Performance by Moving Army Medicine Toward a System for Health. *Journal of Strength and Conditioning Research, 29* Suppl 11, S4-9. <https://doi.org/10.1519/JSC.0000000000001107> |
| 9 | Creamer, M., Wade, D., Fletcher, S., & Forbes, D. (2011). PTSD among military personnel. *International Review of Psychiatry*, *23*(2), 160–165. <https://doi.org/10.3109/09540261.2011.559456> |
| 10 | de Terte, I., & Stephens, C. (2014). Psychological resilience of workers in high‐risk occupations. *Stress and Health: Journal of the International Society for the Investigation of Stress, 30*(5), 353–355. psyh. <https://doi.org/10.1002/smi.2627> |
| 11 | Deuster, P. A., & Silverman, M. N. (2013). Physical fitness: A pathway to health and resilience. *U.S. Army Medical Department Journal*, 24–35. |
| 12 | Dutra, C. C. D., & Rocha, H. S. (2021). Religious Support as a Contribution to Face the Effects of Social Isolation in Mental Health During the Pandemic of COVID-19. *Journal of Religion and Health, 60*(1), 99–111. <https://doi.org/10.1007/s10943-020-01140-2> |
| 13 | Howell, G. (2020). Improving Readiness: Preventive Maintenance of the Human Operating System that Drives Readiness and Lethality. *Military Medicine, 185*(Suppl 3), 12–16. <https://doi.org/10.1093/milmed/usaa162> |
| 14 | King, H. C., Spritzer, N., Eadens, P., & Fisak, J. (2020). Strengthening Resiliency During Pacific Partnership Missions: Leadership Matters. *Military Medicine, 185*(9–10), 417–419. <https://doi.org/10.1093/milmed/usaa086> |
| 15 | Lietz, C. A., Stromwall, L. K., & Carlson, B. E. (2013). Women service members and family reintegration. *Families in Society, 94*(3), 186–193. psyh. <https://doi.org/10.1606/1044-3894.4314> |
| 16 | Masten, A. S. (2013). Competence, risk, and resilience in military families: Conceptual commentary. *Clinical Child and Family Psychology Review, 16*(3), 278–281. <https://doi.org/10.1007/s10567-013-0150-2> |
| 17 | Miller, L. (2008). Stress and resilience in law enforcement training and practice. *International Journal of Emergency Mental Health, 10*(2), 109–124. |
| 18 | Nindl, B. C., Billing, D. C., Drain, J. R., Beckner, M. E., Greeves, J., Groeller, H., Teien, H. K., Marcora, S., Moffitt, A., Reilly, T., Taylor, N. A. S., Young, A. J., & Friedl, K. E. (2018). Perspectives on resilience for military readiness and preparedness: Report of an international military physiology roundtable. *Journal of Science and Medicine in Sport, 21*(11), 1116–1124. <https://doi.org/10.1016/j.jsams.2018.05.005> |
| 19 | Pak, C. (2019). The Downside Risk of Failing to Transcend Extraordinarily Negative Experiences: A Narrative Analysis. *Military Medicine, 184*(1–2), e110–e117. <https://doi.org/10.1093/milmed/usy155> |
| 20 | Rocklein-Kemplin, K., Paun, O., Sons, N., & Brandon, J. W. (2018). The Myth of Hyperresilience Evolutionary Concept Analysis of Resilience in Special Operations Forces. *Journal of Special Operations Medicine : A Peer Reviewed Journal for SOF Medical Professionals, 18*(1), 54–60. |
| 21 | Rothstein, M. G., McLarnon, M. J. W., & King, G. (2016). The role of self-regulation in workplace resiliency. *Industrial and Organizational Psychology: Perspectives on Science and Practice, 9*(2), 416–421. psyh. <https://doi.org/10.1017/iop.2016.32> |
| 22 | Thomas, K. H. (2016). Warrior faith: A Marine’s lesson in religion, health, and healing. *Social Work & Christianity, 43*(3), 109–123. psyh. |
| 23 | Wang, J. Y., Glover, W. J., Rhodes, A. M., & Nightingale, D. (2013). A conceptual model of the psychological health system for U.S. active duty service members: An approach to inform leadership and policy decision making. *Military Medicine, 178*(6), 596–606. <https://doi.org/10.7205/MILMED-D-12-00429> |
| 24 | Wells, T. S., Miller, S. C., Adler, A. B., Engel, C. C., Smith, T. C., & Fairbank, J. A. (2011). Mental health impact of the Iraq and Afghanistan conflicts: A review of US research, service provision, and programmatic responses. *International Review of Psychiatry,* 23(2), 144–152. <https://doi.org/10.3109/09540261.2011.558833> |
| 25 | Wooten, N. R. (2013). A bioecological model of deployment risk and resilience. *Journal of Human Behavior in the Social Environment, 23*(6), 699–717. psyh. <https://doi.org/10.1080/10911359.2013.795049> |
| **IV. No focus on psychological resilience (n = 17)** | |
| 1 | Anestis, J. C., Harrop, T. M., Green, B. A., & Anestis, M. D. (2017). Psychopathic personality traits as protective factors against the development of post-traumatic stress disorder symptoms in a sample of national guard combat veterans. *Journal of Psychopathology and Behavioral Assessment, 39*(2), 220–229. psyh. <https://doi.org/10.1007/s10862-017-9588-8> |
| 2 | Delahaij, R., Kamphuis, W., & van den Berg, C. E. (2016). Keeping engaged during deployment: The interplay between self-efficacy, family support, and threat exposure. *Military Psychology, 28*(2), 78–88. psyh. <https://doi.org/10.1037/mil0000098> |
| 3 | Eisen, S. V., Schultz, M. R., Glickman, M. E., Vogt, D., Martin, J. A., Osei-Bonsu, P. E., Drainoni, M.-L., & Elwy, A. R. (2014). Postdeployment resilience as a predictor of mental health in operation enduring freedom/operation iraqi freedom returnees. *American Journal of Preventive Medicine, 47*(6), 754–761. <https://doi.org/10.1016/j.amepre.2014.07.049> |
| 4 | Frank, C., Lee, J. E. C., & Zamorski, M. A. (2021). Family snapshot: Characteristics of the economic and social environment and their associations with mental health in Canadian military personnel with a history of deployment. *Current Psychology: A Journal for Diverse Perspectives on Diverse Psychological Issues, 40*(9), 4394–4405. psyh. <https://doi.org/10.1007/s12144-019-00357-z> |
| 5 | Hom, M. A., de Terte, I., Bennett, C., & Joiner, T. E. (2020). Resilience and attitudes toward help-seeking as correlates of psychological well-being among a sample of New Zealand Defence Force personnel. *Military Psychology, 32*(4), 329–340. psyh. <https://doi.org/10.1080/08995605.2020.1754148> |
| 6 | Israel-Cohen, Y., Kaplan, O., Noy, S., & Kashy-Rosenbaum, G. (2016). Religiosity as a Moderator of Self-Efficacy and Social Support in Predicting Traumatic Stress Among Combat Soldiers. *Journal of Religion and Health, 55*(4), 1160–1171. <https://doi.org/10.1007/s10943-016-0187-x> |
| 7 | Kamphuis, W., Delahaij, R., & de Vries, T. A. (2021). Team Coping: Cross-Level Influence of Team Member Coping Activities on Individual Burnout. *Frontiers in Psychology, 12*, 711981. <https://doi.org/10.3389/fpsyg.2021.711981> |
| 8 | Mantua, J., Bessey, A. F., & Sowden, W. J. (2020). Poor Subjective Sleep Quality Is Associated with Poor Occupational Outcomes in Elite Soldiers. *Clocks & Sleep, 2*(2), 182–193. <https://doi.org/10.3390/clockssleep2020015> |
| 9 | Moore, B. A., Hale, W. J., Judkins, J. L., Lancaster, C. L., Baker, M. T., Isler, W. C., & Peterson, A. L. (2020). Air Force Medical Personnel: Perspectives Across Deployment. *Military Medicine, 185*(9–10), e1632–e1639. <https://doi.org/10.1093/milmed/usaa115> |
| 10 | Mota, N. P., Medved, M., Whitney, D., Hiebert-Murphy, D., & Sareen, J. (2013). Protective factors for mental disorders and psychological distress in female, compared with male, service members in a representative sample. *Canadian Journal of Psychiatry. Revue Canadienne de Psychiatrie, 58*(10), 570–578. <https://doi.org/10.1177/070674371305801006> |
| 11 | Nakkas, C., Annen, H., & Brand, S. (2016). Psychological distress and coping in military cadre candidates. Neurop*sychiatric Disease and Treatment, 12*, 2237–2243. <https://doi.org/10.2147/NDT.S113220> |
| 12 | Parsloe, L., Jones, N., Fertout, M., Luzon, O., & Greenberg, N. (2014). Rest and recuperation in the UK Armed Forces. *Occupational Medicine, 64*(8), 616–621. <https://doi.org/10.1093/occmed/kqu119> |
| 13 | Schaefer, H., Farina, A., Cotting, D., Proctor, E., Cook, C., & Lerner, R. (2022). The Benefits and Liabilities of Risk-Taking Propensity and Confidence at the US Military Academy. *Armed Forces & Society, 48*(2), 410–439. <https://doi.org/10.1177/0095327X20973373> |
| 14 | Schneider, J. C., Hendrix-Bennett, F., Beydoun, H. A., & Johnstone, B. (2021). A Retrospective Study of Demographic, Medical, and Psychological Predictors of Readiness in Service Members With Mild Traumatic Brain Injury. *Military Medicine, 186*(3–4), e401–e409. <https://doi.org/10.1093/milmed/usaa274> |
| 15 | Tornero-Aguilera, J. F., Pelarigo, J. G., & Clemente-Suárez, V. J. (2019). Psychophysiological Intervention to Improve Preparedness in Military Special Operations Forces. *Aerospace Medicine and Human Performance, 90*(11), 953–958. <https://doi.org/10.3357/AMHP.5385.2019> |
| 16 | Trachik, B., Tucker, R. P., Ganulin, M. L., Merrill, J. C., LoPresti, M. L., Cabrera, O. A., & Dretsch, M. N. (2020). Leader provided purpose: Military leadership behavior and its association with suicidal ideation. *Psychiatry Research, 285*, 112722. <https://doi.org/10.1016/j.psychres.2019.112722> |
| 17 | Welsh, J. A., Olson, J. R., & Perkins, D. F. (2019). Gender differences in post-deployment adjustment of Air Force personnel: The role of wartime experiences, unit cohesion, and self-efficacy. *Military Medicine, 184*(1–2), e229–e234. psyh. <https://doi.org/10.1093/milmed/usy261> |
| **V. Wrong sample (n = 15)** | |
| 1 | Abu-Ras, W., & Hosein, S. (2015). Understanding resiliency through vulnerability: Cultural meaning and religious practice among Muslim military personnel. *Psychology of Religion and Spirituality, 7*(3), 179–191. psyh. <https://doi.org/10.1037/rel0000017> |
| 2 | An, E., Nolty, A. A. T., Amano, S. S., Rizzo, A. A., Buckwalter, J. G., & Rensberger, J. (2020). Heart Rate Variability as an Index of Resilience. *Military Medicine, 185*(3–4), 363–369. <https://doi.org/10.1093/milmed/usz325> |
| 3 | Bartone, P. T., Hystad, S. W., Eid, J., & Brevik, J. I. (2012). Psychological hardiness and coping style as risk/resilience factors for alcohol abuse. *Military Medicine, 177*(5), 517–524. <https://doi.org/10.7205/milmed-d-11-00200> |
| 4 | Conway, B., Hammermeister, J., Briggs, L., Young, J., & Flynn, C. (2016). An alternative path for academic success: Evaluating the role of mental skills in an English composition course. *Journal of College Student Development, 57*(3), 321–325. psyh. <https://doi.org/10.1353/csd.2016.0040> |
| 5 | Dretsch, M. N., Neff, D., Caserta, R., Deagle, E., Hoge, C. W., & Adler, A. B. (2020). Rates of Behavioral Health Conditions and Health Risk Behaviors in Operators and Support Personnel in U.S. Special Operations Forces. *Psychiatry, 83*(4), 358–374. <https://doi.org/10.1080/00332747.2020.1768787> |
| 6 | Eleazer, J. R., Marchant, L., Kizewski, A., Gross, G., Warren, A., & McCubbin, L. (2023). ‘We’ve been here all along:’ The collective resilience of transgender and gender diverse US service members. *Psychological Services, 20*(2), 382–396. psyh. <https://doi.org/10.1037/ser0000721> |
| 7 | Gillispie, S. K., Britt, T. W., Burnette, C. M., & McFadden, A. C. (2016). Employee mental health treatment seeking: Perceptions of responsibility and resilience. *Journal of Workplace Behavioral Health, 31*(1), 1–18. psyh. <https://doi.org/10.1080/15555240.2015.1100519> |
| 8 | Hoorelbeke, K., Van den Bergh, N., Wichers, M., & Koster, E. H. W. (2019). Between vulnerability and resilience: A network analysis of fluctuations in cognitive risk and protective factors following remission from depression. *Behaviour Research and Therapy, 116*, 1–9. <https://doi.org/10.1016/j.brat.2019.01.007> |
| 9 | Johnson, D. C., Polusny, M. A., Erbes, C. R., King, D., King, L., Litz, B. T., Schnurr, P. P., Friedman, M., Pietrzak, R. H., & Southwick, S. M. (2011). Development and initial validation of the Response to Stressful Experiences Scale. *Military Medicine, 176*(2), 161–169. <https://doi.org/10.7205/milmed-d-10-00258> |
| 10 | Leners, C., Sowers, R., Quinn Griffin, M. T., & Fitzpatrick, J. J. (2014). Resilience and professional quality of life among military healthcare providers. *Issues in Mental Health Nursing, 35*(7), 497–502. <https://doi.org/10.3109/01612840.2014.887164> |
| 11 | Pickering, M. A., Hammermeister, J., Ohlson, C., Holliday, B., & Ulmer, G. (2010). An exploratory investigation of relationships among mental skills and resilience in Warrior Transition Unit cadre members. *Military Medicine, 175*(4), 213–219. <https://doi.org/10.7205/milmed-d-09-00059> |
| 12 | Reed-Fitzke, K., Ferraro, A. J., Duncan, J. M., Wojciak, A. S., Hamilton, A., & Pippert, H. D. (2023). Resilience in Army STARRS: Evaluating psychometrics of a multi-dimensional resilience measure. *Military Psychology, 35*(6), 521–528. <https://doi.org/10.1080/08995605.2022.2131187> |
| 13 | Wallace, J. R. (2016). Field test of a peer support pilot project serving federal employees deployed to a major disaster. *Social Work & Christianity, 43*(1), 127–141. psyh. |
| 14 | Ward, R. N., Erickson, A. J., Carlson, K. J., & Yalch, M. M. (2023). The role of unit cohesion and perceived resilience in substance use disorder. *Military Psychology*. psyh. <https://doi.org/10.1080/08995605.2023.2189861> |
| 15 | Zlotnick, C., Manor-Lavon, I., & Srulovici, E. (2022). Resilience in late adolescence/young adulthood: Rising to the occasion? *Military Psychology*. psyh. <https://doi.org/10.1080/08995605.2021.2017190> |
| **VI. Related concept of resilience (n = 11)** | |
| 1 | Bartone, P. T., & Bowles, S. V. (2020). Coping with recruiter stress: Hardiness, performance and well-being in US Army recruiters. *Military Psychology, 32*(5), 390–397. psyh. <https://doi.org/10.1080/08995605.2020.1780061> |
| 2 | de Souza, Y. R., & Feitosa, F. B. (2015). The impact of coping strategies on the operations in the jungle course of the Brazilian army. *Military Psychology, 27*(6), 348–353. psyh. <https://doi.org/10.1037/mil0000084> |
| 3 | Haufler, A. J., Lewis, G. F., Davila, M. I., Westhelle, F., Gavrilis, J., Bryce, C. I., Kolacz, J., Granger, D. A., & McDaniel, W. (2017). Biobehavioral Insights into Adaptive Behavior in Complex and Dynamic Operational Settings: Lessons learned from the Soldier Performance and Effective, Adaptable  Response Task. *Frontiers in Medicine, 4*, 217. <https://doi.org/10.3389/fmed.2017.00217> |
| 4 | Kelly, P. J., Cheng, A.-L., Berkel, L. A., & Nilsson, J. (2016). Perceptions of Individual and Family Functioning Among Deployed Female National Guard Members. *Journal of Family Nursing, 22*(3), 419–434. <https://doi.org/10.1177/1074840716642791> |
| 5 | Krauss, S. W., Russell, D. W., Kazman, J. B., Russell, C. A., Schuler, E. R., & Deuster, P. A. (2019). Longitudinal effects of deployment, recency of return, and hardiness on mental health symptoms in US Army combat medics. *Traumatology, 25*(3), 216–224. psyh. <https://doi.org/10.1037/trm0000173> |
| 6 | Kulak, J. A., Homish, D. L., Hoopsick, R. A., Fillo, J., Bartone, P. T., & Homish, G. G. (2021). Hardiness protects against problematic alcohol use in male, but not female, soldiers. *Psychological Services, 18*(3), 426–432. <https://doi.org/10.1037/ser0000409> |
| 7 | Nordmo, M., Hystad, S. W., Sanden, S., & Johnsen, B. H. (2017). The effect of hardiness on symptoms of insomnia during a naval mission. *International Maritime Health, 68*(3), 147–152. <https://doi.org/10.5603/IMH.2017.0026> |
| 8 | Rybakovaitė, J., Bandzevičienė, R., & Poškus, M. S. (2021). The impact of psychological hardiness on soldiers’ engagement and general health: The mediating role of need satisfaction. *Current Psychology: A Journal for Diverse Perspectives on Diverse Psychological Issues*. psyh. <https://doi.org/10.1007/s12144-021-01371-w> |
| 9 | Skomorovsky, A., & Stevens, S. (2013). Testing a resilience model among Canadian Forces recruits. *Military Medicine, 178*(8), 829–837. <https://doi.org/10.7205/MILMED-D-12-00389> |
| 10 | Thomassen, Å. G., Hystad, S. W., Johnsen, B. H., Johnsen, G. E., & Bartone, P. T. (2018). The effect of hardiness on PTSD symptoms: A prospective mediational approach. *Military Psychology, 30*(2), 142–151. psyh. <https://doi.org/10.1080/08995605.2018.1425065> |
| 11 | Thomassen, Å. G., Hystad, S. W., Johnsen, B. H., Johnsen, G. E., Laberg, J. C., & Eid, J. (2015). The combined influence of hardiness and cohesion on mental health in a military peacekeeping mission: A prospective study. *Scandinavian Journal of Psychology, 56*(5), 560–566. <https://doi.org/10.1111/sjop.12235> |
| **VII. Resiliene training (n = 1)** | |
| 1 | Fikretoglu, D., Liu, A., Nazarov, A., & Blackler, K. (2019). A group randomized control trial to test the efficacy of the Road to Mental Readiness (R2MR) program among Canadian military recruits. *BMC Psychiatry*, *19*(1), 326. <https://doi.org/10.1186/s12888-019-2287-0> |
