# Supplementary material for: Soldiers’ resilience? A systematic review of micro-, meso-, and macro-level resilience factors in the military
Source: Front Psychol. 2026 Jul 22;17:1857328. doi: 10.3389/fpsyg.2026.1857328 (PMC13437443; doi:10.3389/fpsyg.2026.1857328)
Supplement: Supplementary file 5 [file Table_5.docx]

**Supplementary File D - Quality Assessment**

**Supplementary Table D1: Quality Assesment**

| **Study** | **Appraisal Tool** | **Overall Quality** |
| --- | --- | --- |
| Adler et al. (2023) | JBI | High |
| Cai et al. (2017) | JBI | Moderate |
| Charbonneau (2019) | JBI | High |
| Reyes et al. (2020) | JBI | Moderate |
| Rice & Liu (2016) | JBI | High |
| Valladares-Garrido et al. (2022) | JBI | High |
| Yurgil et al. (2024) | CASP Cohort Study Checklist | Moderate |
| Abraham et al. (2018) | CASP Qualitative Checklist | High |
| Doody et al. (2022) | CASP Qualitative Checklist | High |

**Supplementary Table D2: Item-level appraisal – JBI Checklist for Analytical Cross Sectional Studies**

|  | **Q1** | | **Q2** | | **Q3** | | **Q4** | | **Q5** | | **Q6** | | **Q7** | | **Q8** | | **Comment** |
| --- | --- | --- | --- | --- | --- | --- | --- | --- | --- | --- | --- | --- | --- | --- | --- | --- | --- |
| *Screening Person* | *1* | *2* | *1* | *2* | *1* | *2* | *1* | *2* | *1* | *2* | *1* | *2* | *1* | *2* | *1* | *2* |  |
| Adler et al. (2023) | y | y | y | y | y | y | y | y | y | y | y | y | y | u | y | y | Self-constructed scales were used for assessing wellness-checks. |
| Cai et al. (2017) | y | y | y | y | y | y | na | na | u | n | n | n | y | y | y | y | No confounding factors. |
| Charbonneau (2019) | y | y | y | y | y | y | y | y | y | y | y | y | y | y | y | y | - |
| Reyes et al. (2020) | y | y | y | y | y | y | na | na | u | u | u | u | y | y | y | y | No confounding factors. |
| Rice & Liu (2016) | y | y | y | y | y | y | y | y | y | y | y | y | y | y | y | y | - |
| Valladares-Garrido et al. (2022) | y | y | y | y | y | y | y | y | y | y | y | y | y | y | y | y | - |

*Note*. y = yes; no = no; u = unclear; n.a. = not applicable.

**Supplementary Table D3: Item-level appraisal – CASP Qualitative Checklist**

|  | **Q1** | | **Q2** | | **Q3** | | **Q4** | | **Q5** | | **Q6** | | **Q7** | | **Q8** | | **Q9** | | **Q10** | | **Comment** |
| --- | --- | --- | --- | --- | --- | --- | --- | --- | --- | --- | --- | --- | --- | --- | --- | --- | --- | --- | --- | --- | --- |
| *Screening Person* | *1* | *2* | *1* | *2* | *1* | *2* | *1* | *2* | *1* | *2* | *1* | *2* | *1* | *2* | *1* | *2* | *1* | *2* | *1* | *2* |  |
| Abraham et al. (2018) | y | y | y | y | y | y | y | y | y | y | n | c | y | y | y | y | y | y | y | Y | Limited discussion of researcher-participant relationship |
| Doody et al. (2022) | y | y | y | y | c | c | y | y | y | y | y | y | y | y | y | y | y | y | y | y | Limited justification of study design and snowball sampling strategy. |

*Note*. y = yes; no = no; c = can’t tell.

**Supplementary Table D4: Item-level appraisal – CASP Cohort Study Checklist**

|  | **Q1** | | **Q2** | | **Q3** | | **Q4** | | **Q5a** | | **Q5b** | | **Q6a** | | **Q6b** | | **Q7** | | **Q8** | | **Q9** | | **Q10** | | **Q11** | | **Q12** | | **Comment** |
| --- | --- | --- | --- | --- | --- | --- | --- | --- | --- | --- | --- | --- | --- | --- | --- | --- | --- | --- | --- | --- | --- | --- | --- | --- | --- | --- | --- | --- | --- |
| *Screening Person* | *1* | *2* | *1* | *2* | *1* | *2* | *1* | *2* | *1* | *2* | *1* | *2* | *1* | *2* | *1* | *2* | *1* | *2* | *1* | *2* | *1* | *2* | *1* | *2* | *1* | *2* | *1* | *2* |  |
| Yurgil et al. (2024) | y | y | y | c | y | y | y | y | y | y | y | y | c | c | c | c | y |  | y |  | y | y | y | y | y | y | y | y | Insufficient reporting of follow-up duration and completeness. |

*Note*. y = yes; no = no; c = can’t tell; n.a. = not applicable.
