# Supplementary material for: Soldiers’ resilience? A systematic review of micro-, meso-, and macro-level resilience factors in the military
Source: Front Psychol. 2026 Jul 22;17:1857328. doi: 10.3389/fpsyg.2026.1857328 (PMC13437443; doi:10.3389/fpsyg.2026.1857328)
Supplement: Supplementary file 6 [file Table_6.docx]

**Supplementary File C – Coding Rules and Examples**

**Table C1. Coding Rules**

| **Theme** | **Definition** | **Coding Rule** |
| --- | --- | --- |
| Micro level resilience factors | | |
| Interpersonal resources | Qualities, abilities or experiences that arise within the interaction between individuals. | Primary source and mechanism of influence within the individual person. |
| Intrapersonal resources | Individual qualities, abilities or experiences within a person that enable one’s capability and efficacy. | Individual capacities that support social functioning, relationships or helping behaviours. |
| Knowledge | Acquisition and integration of information, experience and abilities that enables a person to gain a deeper understanding of the military and its tasks. | Knowledge learning or experience primarily acquired and utilized by the individual. |
| Military identity | Believes and strong (emotional) attachment with the military that influences one’s behaviour. | Identification with military service, values or a sense of purpose related to military roles. |
| Meso level resilience factors | | |
| Social environment | Several factors of social relationships and their interplay that influence the social interaction between individuals, teams and units positively. | Primary mechanism of influence through social interaction, cohesion, support or leadership processes. |
| Training | Various informal elements through social interaction (knowledge sharing) that provide military personnel with the relevant information, experience and abilities and prepare them to fulfil their tasks positively. | Knowledge, skills or experiences primarily transmitted through interpersonal or team-based interactions. |
| Macro level resilience factors | | |
| Knowledge | Access to information that enables a person to gain a deeper understanding of the military and its tasks. | Access to information primarily facilitated by organizational structures or procedures. |
| Organizational framework | Structures and processes within the military organisation containing various rules that enable the organisation to function positively. | Factors are embedded in the military organization. |
| Training | Various official elements that provide military personnel with the relevant information, experience and abilities and prepare them to fulfil their tasks positively. | Training is planned, organized, and implemented at the institutional level. |

**Table C2. Examples of ambiguous factor classification**

| **Resilience factor** | **Final level** | **Ambiguity** | **Rationale** |
| --- | --- | --- | --- |
| Awareness of the military culture | Micro | Meso | An individual’s understanding of military culture rather than the culture itself. |
| Social bonding behaviour | Micro | Meso | An individual’s ability to establish social connections. |
| Social bonding with the crew | Meso | Micro | Emerges through interactions and relationships within the social environment. |
| To be informed | Macro | Micro, Meso | Organisational provision of information rather than interpersonal information exchange or intrapersonal knowledge. |
| Leadership behaviour | Meso | Meso, macro | Leadership behaviour rather than organizational leadership principles or guidelines. |

**Table C3. Resilience factor assigned to multiple levels**

| **Resilience factor** | **Level** | **Rational** |
| --- | --- | --- |
| Exchanging/sharing knowledge and skills | Micro | Classified as micro when described as an individual behaviour of active expertise sharing. |
|  | Meso | Classified as meso when described as a reciprocal team-based learning process |
